# New Advertisers

**Published:** 1919-04

**Authors:** 


### OUR NEW ADVERTISERS.

### The Temple Sanitarium.

The Temple Sanitarium will have a page in each issue of THE TEXAS MEDICAL JOURNAL hereafter. It has long been one of the most popular sanitariums of the State of Texas, but it has been growing at such a rapid rate lately that it is now classed among the large institutions of the country. It has frequently been styled the Mayo Clinic of the South. It is doubtful if in the whole country a stronger hospital staff can be found, or if anywhere better surgical and medical attention can be had. The business is growing so rapidly that the buildings are spreading over several city blocks. The sanitarium now occupies some seventeen buildings and more will be ereted right away to accommodate the growth of the institution.

### Dr. G. H. Sherman.

Sherman's Bacterial Vaccines are to be advertised regularly in THE TEXAS MEDICAL JOURNAL, the first announcement appearing this month. Dr. G. H. Sherman began the manufacture of bacterial vaccines in Detroit, Michigan on a very small scale, but the merit of his products have been such that he now occupies a large, three-story building of his own, and his business extends practically over the entire country. Those who patronize him may be sure of getting the best and of having a fair deal.

### Henry Pharmacal Company.

The Henry Pharmacal Company, of St. Louis, were advertising in this journal when the war made it difficult to get supplies. They are back again and this time to stay, so physicians will do well to watch their announcements in our advertising columns from month to month. Their advertising is always attractive and distinctive as well as interesting to the profession.

### Campho-Phenique Co.

The Campho-Phenique Co., of St. Louis, again presents the merits of Campho-Phenique, the well-known antiseptic dressing, for the consideration of physicians. Campho-Phenique is put out in liquid and powder form and also as an ointment, and its marked germicidal power is recognized by those who are acquainted with its properties. It has won its way to the confidence of practitioners.

### Torbett Sanitarium.

This popular Sanatorium, Hotel and Bath House, of which Dr. J. W. Torbett is superintendent, has a new card in THE TEXAS MEDICAL JOURNAL. Marlin has become one of the most famous health resorts of the United States and its fame rests in large measure on the remarkable success of the Torbett Sanatorium and its fine staff of specialists.

---

### Platt's Chlorides.

Platt's Chlorides have been popular for deodorizing sick rooms, bed-pans, urinals and every place that might without its use have an objectionable odor. Physicians who look after keeping the sick room sweet recommend its use and many hospitals use it exclusively as a deodorizer. The advertising will appear regularly in THE TEXAS MEDICAL JOURNAL hereafter. When you think of an odorless disinfectant you think of Platt's Chlorides.

---

### Hotel Raleigh.

When the Texas State Medical Convention goes to Waco May 13-15 its headquarters will be at the Hotel Raleigh, which has a half page advertisement in this number of the Texas Medical Journal. The Raleigh has over 200 roooms, and is one of the most modernly constructed and conducted hotels in the South. It will be the center of things during convention week, as it is at other Waco conventions.

---

### Waco Drug Co.

The Waco Drug Company uses space in this issue of the Texas Medical Journal to invite physicians and surgeons when in Waco to visit their place of business. It is one of Texas' largest wholesale drug stores and is a place of interest to doctors. Call to see them when in Waco.

---

### Hotel Waco.

J. D. Adams is one of Waco's oldest hotel men. He invites you to stop with him at Hotel Waco while attending the convention next month. Everything is good at Hotel Waco.

---

### St. Charles Hotel.

The announcement made by the St. Charles Hotel in this issue of THE TEXAS MEDICAL JOURNAL that it will have rooms at from $1.00 up will be good news to doctors who want a good medium-priced hotel.

